# Cultivating organizational performance through the performance measurement systems: Role of psychological empowerment and creativity

**DOI:** 10.3389/fpsyg.2023.1116617

**Published:** 2023-03-22

**Authors:** Lu Zhang, Dalgon Kim, Shusheng Ding

**Affiliations:** ^1^Business School, Ningbo University, Ningbo, China; ^2^Business School, Gyeongsang National University, Jinju, Republic of Korea

**Keywords:** interactive performance measurement system use, psychological empowerment, creativity, organizational performance, structural equation modelling

## Abstract

Interactive performance measurement systems (PMSs) play a critical role in shaping individual behavior and performance. To identify the underlying mechanism of how PMSs enhance organizational performance, a proposed model was constructed to investigate psychological empowerment and employee creativity as possible mediating variables. Based on a sample of 211 managers from Chinese organizations, a partial least squares structural equation modelling (PLS-SEM) approach was used to examine the mediating effect presented in the aim. Interactive use of PMS has a positive and direct impact on psychological empowerment. Psychological empowerment positively influences creativity, which, in turn, positively influences organizational performance. The findings also show that psychological empowerment and creativity mediate the impacts of interactive use of PMS on organizational performance. Our study highlights the role of PMSs, and how to use them interactively in turbulent environments. Particularly, we demonstrate that interactive use of PMS is important for facilitating a manager’s sense of psychological empowerment and fostering creativity, which, in turn, contributes to better performance and greater competitive advantages.

## 1. Introduction

Creativity and psychological empowerment have become popular issues in today’s turbulent and competitive business environment (Amabile, 1988; Zhou and George, 2001). Based on resource-based theory (RBT), creativity is an intangible resource embedded within a firm that can provide a competitive advantage (Barney, 1991; Im and Workman, 2004). Recent research has indicated that psychological empowerment plays a key role in facilitating individual creativity (Amabile et al., 1996; Zhang and Bartol, 2010; Moulang, 2015) because psychological empowerment acts as an internal stimulator, which allows individuals to perceive their own ability to skillfully perform their work activities (Spreitzer, 1995). This cognition of psychological empowerment contributes to an improvement in individual performance outcomes (Zhang and Bartol, 2010; Dust et al., 2018; Malik et al., 2020). Creativity refers to the generation of novel and useful ideas (Amabile et al., 1996), and psychological empowerment refers to the individual perceptions of meaning, competence, self-determination and impact (Spreitzer, 1995).

Performance measurement systems (PMSs) play a crucial role in enhancing an individual’s motivation. Researchers have agreed on the effect that PMS has on motivation from the perspective of individual behavior (Godener and Söderquist, 2004; Webb, 2004; Decoene and Bruggeman, 2006; Hall, 2008; Franco-Santos et al., 2012; Appuhami, 2019). These studies demonstrate that PMS as an effective communication mechanism enhances participation, dialogue and feedback, and contribute to stimulating motivation.

PMS is “a cybernetic tool used in planning, reporting and monitoring, which utilises a mix of financial and non-financial metrics to quantify performance” (Simons, 1995; Henri, 2006b; Cäker and Siverbo, 2018). Moreover, PMS is used by organizations to stimulate and guide learning, innovation and creativity (Simons, 1995; Henri, 2004). Existing literature has categorized the purposes of PMSs into two. First, diagnostic use of PMSs is the formal information system that managers use to monitor organizational outcomes and correct deviations from preset standards of performance. Second, interactive use of PMS is a catalyst forcing the firm to monitor changing market dynamics and inspire discussions about data, assumptions and action plans (Simons, 1995). Diagnostic use clearly emphasises control and efficiency, whilst interactive use focuses attention on strategic uncertainties, facilitates debate, dialogue and creative thinking and stimulates emergent strategies (Simons, 1995; Henri, 2006b).

The role of interactive PMS usage is more apparent in turbulent environments, where they are beneficial for enhancing empowerment (Marginson et al., 2014) whilst supporting innovation and creativity (Henri, 2006a; Müller-Stewens et al., 2020). Interactive PMS use enables the construction of a participative environment that values openness and constructive feedback/conversations. In this participative climate, organizational members are more likely to dissent, take on more risks, share information and propose novel ideas (Simons, 1995). Conversely, the autonomy and psychological states of participants may be threatened if the quality of their effort or thinking is challenged publicly (Simons, 1995).

We aim to explain the mechanism by which interactive PMS use affects organizational performance *via* the mediation effect of psychological empowerment and creativity. Our work extends previous literature on the effect that PMSs have on individual behavior by examining the implications of the relationship between PMS, individual behavior and performance. Empirical studies such as Hall (2008) and Marginson et al. (2014) have suggested that a diverse set of performance measures can have a positive effect on empowerment. Along those lines, Moulang (2015) and Speklé et al. (2017) have also suggested the positive effect of interactive PMS use on creativity through psychological empowerment. When PMSs are used interactively, continual debate and communication occur throughout the organization, manager empowerment is fostered, which leads to greater manager creativity. Moreover, prior research has highlighted that the role of PMS, as it is the resultant performance measures that yield motivational benefits according to cognitive-based psychological theory (Malina and Selto, 2001; Webb, 2004; Hall, 2008). For instance, Simons (1995) argued that different uses of PMS can generate different psychological consequences. Franco-Santos et al. (2012) have argued that to drive motivation, PMSs should be designed and used in a way that improves the sense of psychological empowerment. Similarly, Hall (2008) concluded that PMS usage can help to improve managers’ cognition and motivation.

Whilst existing literature has focused mainly on the consequences of PMS in terms of organizational capabilities and performance (Chenhall, 2005; Henri, 2006b; Mundy, 2010), they contain little information concerning the effect of PMSs on individual behavior, and little empirical evidence to support the association between PMS, psychological empowerment and creativity. Particularly, the studies have only investigated the impact of PMS on individual’s behavior in terms of role ambiguity, perception of justice, trust, goal commitment and organizational citizenship behavior (Webb, 2004; Burney and Widener, 2007; Burney et al., 2009; Franco-Santos et al., 2012). Comprehension is lacking on how PMS usage influences the psychological empowerment of managers, which, in turn, improves creativity and performance. Furthermore, the present study addresses (Moulang, 2015) call for further study on PMS uses by focusing on managers from different hierarchical levels. Our findings also contribute to PMS literature by utilising data collected from different cultural backgrounds (Koufteros et al., 2014). As PMS is not immune to these effects, economic development and regional differences can pervasively affect firms (Koufteros et al., 2014). Hence, there is a need to explore the association between interactive use of PMS, psychological empowerment, creativity and organizational performance. Specifically, four research questions are investigated: (i) Does an interactive PMS promote an improvement in psychological empowerment? (ii) Does psychological empowerment boost individual creativity? (iii) Does creativity enhance organizational performance? (iv) How does the interactive PMS affect organizational performance *via* psychological empowerment and creativity? Structural equation modelling was used to provide empirical evidence. Survey data were gathered from 211 managers in Chinese firms across the Beijing, Shanghai, Zhejiang and Anhui regions of China, which is one of the world’s most successful emerging economies.

## 2. Theory and hypotheses

### 2.1. Interactive performance measurement systems and psychological empowerment

Psychological empowerment refers to a motivational construct manifested in a set of our cognitions; meaning, competence, self-determination and impact (Spreitzer, 1995). Specifically, meaning indicates the alignment between an individual’s work role and an individual’s ideals or standards. Competence represents one’s own ability to achieve goals. Self-determination refers to the ability to make choices regarding the initiation and regulation of actions. Impact represents the ability to produce intended effects (Deci et al., 1989; Thomas and Velthouse, 1990).

Current research suggested that interactive PMS use positively affects psychological empowerment. Simons (1995) argued that interactive controls create an empowered organization. According to Spreitzer (1996), psychological empowerment determinants comprise relevant support networks, a wide span of control, access to information and a participative work climate. These determinants can be achieved *via* interactive PMS use, which is supported by empirical analyses. For instance, Hall (2008) suggested that a comprehensive PMS can positively affect elements of psychological empowerment. Appuhami (2019) emphasized that PMSs enhance the psychological empowerment of managers by facilitating dialogue at all levels of firms. Marginson et al. (2014) and Speklé et al. (2017) also provided evidence of the psychologically beneficial role played by interactive PMS. Interactive controls provide the formal information conduits needed to transmit learning throughout the organization and thus capture the benefits of individual initiative (Simons, 1995). Using a PMS interactively stimulates the initiative to make individual contributions, and creates a work context that facilitates psychological empowerment. It can empower firms because it provides autonomy support (Speklé et al., 2017), which enhances the sense of psychological empowerment (Spreitzer, 1995; Malik et al., 2020). Thus, we propose:

*Hypothesis 1*: Interactive PMS use positively influences psychological empowerment.

### 2.2. Psychological empowerment and creativity

Creativity can be defined as the production of novel and useful ideas (Woodman et al., 1993; Amabile et al., 1996; Ambrose and Kulik, 1999). Researchers view psychological empowerment as a motivational construct and define it as intrinsic motivation manifested as a set of cognitions (Thomas and Velthouse, 1990; Spreitzer, 1995). Existing studies have supported the notion that intrinsic motivation is one of the most important and powerful influences on creativity (Amabile, 1988; Zhang and Bartol, 2010). Studies by Ambrose and Kulik (1999) and Amabile (1983) have emphasized how closely employee creativity is linked to the motivational process. This demonstrates that psychological empowerment is conceptually highly relevant to creativity.

Empirical studies have proven a positive impact of psychological empowerment on creativity. For instance, Speklé et al. (2017) demonstrated that psychological empowerment positively influences creativity. Moulang (2015) also argued that the cognitions of psychological empowerment positively relate to creativity, claiming that individuals who feel psychologically empowered are more likely to produce creative ideas, thoughts and activities. Similarly, Zhang and Bartol (2010) believed that psychological empowerment impacts creativity by positively affecting an individual’s intrinsic motivation. Other studies have also suggested that feelings of self-efficacy and competence can boost higher creativity (Mumford and Gustafson, 1988; Redmond et al., 1993). Moreover, psychological empowerment has been shown to affect innovative individual behavior (Spreitzer et al., 1999; Chamberlin et al., 2018; Malik et al., 2020). Overall, psychological empowerment as an internal stimulator increases an individual’s orientation to work. This cognition results in new and innovative ideas, enhancing creativity. Therefore, we propose:

*Hypothesis 2*: Psychological empowerment positively influences creativity.

### 2.3. Creativity and organizational performance

Based on RBT, creativity is an intangible resource, which can provide a competitive advantage (Barney, 1991; Im and Workman, 2004). Creativity can lead to product differentiation, which is a crucial determinant of organizational performance (Andrews and Smith, 1996; Song and Parry, 1997; Song and Montoya-Weiss, 2001; Im and Workman, 2004). However, empirical studies on the link between creativity and performance are sparse. Previous studies have suggested that individual-level creativity has an impact on job performance outcomes. For example, Gong et al. (2009) claimed that employee creativity impacts supervisor-rated employee job performance. Oldham and Cummings also argued that there exists a positive correlation between employee creativity and job performance (Gong et al., 2009). Additionally, scholars have also examined the relationship between team creativity and organizational performance. For instance, Sung and Nam (2012) showed that team creativity was a significant predictor of team financial performance. This highlights the importance of team creativity on team performance, which was proven by Im and Workman (2004), who showed the positive effect of creativity on new product performance at the team level (i.e., relative market sales, relative profitability and meeting objectives for customer satisfaction).

Other researchers have also empirically examined the link between firm-level creativity and organizational performance. Specifically, Mikalef and Gupta (2021) found that organizational creativity is directly associated with organizational performance. They suggested that organizational creativity contributes to organizational performance gains. Von nordenflycht (2007) found that creativity results in competitive differentiation, resulting in firm-level success, whilst Weinzimmer et al. (2011) highlighted the direct effect of creativity on organizational performance. Therefore, creativity as a comparative advantage can yield sustaining competitiveness and, thereby, superior financial performance (Hunt and Morgan, 1995). In other words, creativity can provide competitive advantages to a firm and improve organizational performance because it is a strategic resource that is inimitable and non-substitutable (Barney, 1991; Im and Workman, 2004). Accordingly, we propose:

*Hypothesis 3*: Creativity positively influences organizational performance.

*Hypothesis 3-1*: Creativity positively influences financial performance.

*Hypothesis 3-2*: Creativity positively influences non-financial performance.

### 2.4. The mediating role of psychological empowerment

Current scholars emphasized that organizational contexts—open information sharing, participative decision-making and decentralisation—are strongly related to psychological empowerment (Spreitzer, 1996; Seibert et al., 2011). These facilitating factors can be accomplished *via* interactive PMS. By encouraging communication and debate, interactive use of PMSs plays a role in the facilitation of intrinsic task motivation in organizational participants (Simons, 1995; Hall, 2008; Bisbe and Malagueño, 2012). Moreover, researchers have proposed individual creativity as a key outcome of psychological empowerment (Redmond et al., 1993; Spreitzer, 1995; Zhou, 2003). Thomas and Velthouse (1990) also demonstrate that higher levels of creativity have been positively related to perceived choice in one’s actions.

Empirical evidence of the link between interactive PMS use, psychological empowerment and creativity is still relatively lacking (Moulang, 2015; Speklé et al., 2017). Moulang (2015) argued that interactive use of PMS has a positive effect on creativity through psychological empowerment. Speklé et al. (2017) provided support for the theoretical position that interactive PMSs indirectly affect employee creativity *via* psychological empowerment. Appuhami (2019) noted that creativity is indirectly influenced by interactive PMS through the competence dimension of psychological empowerment. Furthermore, other studies suggested that psychological empowerment is seen as a crucial mediating factor in the relationship between interactive PMSs and job performance (Hall, 2008; Marginson et al., 2014). As a result, we expect interactive PMSs to motivate dialogue and discussion, provide adequate information for generating a positive psychological experience (Simons, 1995; Marginson et al., 2014; Speklé et al., 2017), and then facilitate the creative activities within managers’ work role (Otley, 1994; Moulang, 2015). Therefore, we propose:

*Hypothesis 4*: Psychological empowerment mediates the association between interactive PMS use and creativity.

### 2.5. The mediating role of creativity

Based on cognitive evaluation theory, creativity is associated with intrinsic motivation (Amabile et al., 1990). In other words, individual’s intrinsic motivation can yield important work outcomes, particularly on tasks requiring creativity (Gagné and Deci, 2005). Alge et al. (2006) have also highlighted the significance of empowerment, suggesting empowered individuals have the freedom to explore novel ideas, products or processes. This has been confirmed through empirical analysis that has focused on exploring creativity as an outcome of psychological empowerment. For example, Sun et al. (2012) proved that psychological empowerment has a direct effect on individual creativity. Other studies (Zhou, 2003; Moulang, 2015; Speklé et al., 2017) argued that psychological empowerment strongly influenced creativity. In other words, empowerment enables individuals to feel they can achieve their work capably and encourage self-initiation (Conger and Kanungo, 1988; Thomas and Velthouse, 1990), which, in turn, have positive consequences for creativity. Additionally, previous studies also showed that self-determination is an essential component of psychological empowerment, and boosts greater initiative and creativity (Deci and Ryan, 1985).

Creativity is a distinctive and valuable resource, which is critical for firms’ survival and competitiveness (Oldham and Cummings, 1996; Gong et al., 2009). Previous research has mostly demonstrated the antecedents of creativity (Shalley and Gilson, 2004), but few researches have investigated the consequences of creativity. For example, some empirical studies have confirmed that job satisfaction is a mediating variable between psychological empowerment and performance (Ayoub et al., 2018). However, evidence of the influence of psychological empowerment on performance *via* creativity is still relatively scarce, even if researchers have provided evidence that individual creativity enhances innovative work behavior (Volery and Tarabashkina, 2021) and performance outcomes (Weinzimmer et al., 2011; Mikalef and Gupta, 2021). Creativity occurs only when organizational participants are intrinsically motivated (Caniëls et al., 2014), which fosters performance improvement (Im and Workman, 2004; Sung and Nam, 2012). Hence, the link between psychological empowerment and performance is likely to be formed through creativity. Psychological states of empowerment of managers motivate them to produce more creative work (Appuhami, 2019), which sequentially impacts a firm’s ability to achieve performance. Accordingly, we propose:

*Hypothesis 5*: Creativity mediates the association between psychological empowerment and organizational performance.

*Hypothesis 5-1*: Creativity mediates the association between psychological empowerment and financial performance.

*Hypothesis 5-2*: Creativity mediates the association between psychological empowerment and non-financial performance.

## 3. Methods

### 3.1. Participants and procedure

Data for this study were collected using an onsite survey and an online version of the questionnaire (wjx.cn). We targeted high-level executives who had a clear understanding of the ongoing operations and management. In November 2021, the questionnaire was distributed to Master of Business Administration (MBA) students from Ningbo University and leading accounting personnel in Ningbo city. A total of 300 managers participated in our study. After deducting six invalid questionnaires, we received 211 usable responses (70.33% response rate). In organizational sciences literature, there has been some discussion on what an appropriate response rate would be for studies of this nature (Baruch and Holtom, 2008). Based on their recommendation, we conclude that the response rate of our study was also acceptable for data analysis.

To address common method variance (CMV) concerns, we followed ex-ante and ex-post remedies recommended by Podsakoff et al. (2003). The questionnaire was pre-tested to ensure it was designed at a level the respondents could comprehend. Participants were assured in the cover letter that all surveys were anonymous, non-commercial and that the data would only be used for academic purposes. The survey title was abstract and did not give any idea of the research questions. To avoid similarities or redundancy in the research questions, we dispersed similar research questions throughout the survey. We reminded respondents of how research can benefit them and encouraged respondents to read each question carefully. Ex-post, we adopted Harman’s one-factor test to assess CMV. Both tests show that CMV is of no general concern in this study (Podsakoff et al., 2003).

We examined the presence of non-response bias by comparing early and late respondents based on the return time. The mean values of each variable were compared to identify whether early respondents differed significantly from the late respondents (response after a reminder). The results of a student’s t-test showed no significant differences between the two groups for interactive PMS use, psychological empowerment, creativity and financial/non-financial performance. Thus, no evidence for a bias was found.

### 3.2. Measures

#### 3.2.1. Interactive use of performance measurement systems

Interactive use of PMS was measured using Henri (2006b) 5-item scale. Consistent with Henri (2006b) suggestion, this measurement is justified by its development, based on accounting control theory. Sample items included ‘Enable discussion in meetings of superiors, subordinates, and peers’ and ‘Enable the firm to focus on critical success factors.’ Respondents were asked to rate these five items on a 7-point Likert-type scale (1 = *strongly disagree*, 7 = *strongly agree*). The Cronbach’s α score for this scale was 0.909.

#### 3.2.2. Psychological empowerment

Psychological empowerment was assessed using Spreitzer (1995) 12-item scale. This instrument is a four-dimensional measure. According to Hall (2008), it is more appropriate to identify each empowerment dimension with three items. Sample items included ‘The work I do is meaningful to me’ and ‘My impact on what happens in my work area is large.’ Respondents were required to evaluate these 12 items on a 7-point Likert-type scale (1 = *strongly disagree*, 7 = *strongly agree*). The Cronbach’s α score for this scale was 0.917.

#### 3.2.3. Creativity

Creativity was assessed using Zhou and George (2001) 13-item scale. According to their suggestion and recent literature on creativity, this instrument is more appropriate to measure creativity at the individual level. Sample items included ‘I research new technologies, processes, techniques, and/or product ideas’ and ‘I come up with creative solutions to problems.’ Respondents were asked to rate these 13 items on a 7-point Likert-type scale (1 = *strongly disagree*, 7 = *strongly agree*). The Cronbach’s α score for this scale was 0.961.

#### 3.2.4. Organizational performance

Organizational performance refers to the degree of goal attainment along several dimensions relative to competitive enterprises, both financial and non-financial. We measured organizational performance using an 8-item scale adopted from Abernethy and Brownell (1999) and Ittner et al. (2003). Respondents were required to evaluate these eight items on a 7-point Likert-type scale (1 = *strongly disagree*, 7 = *strongly agree*). Four indicators were selected from a financial perspective: (i) sales growth rate; (ii) operating profits rate; (iii) annual profits and (iv) return on assets (ROA). The Cronbach’s α for this scale was 0.927. Four indicators were selected from a non-financial perspective: (i) market share; (ii) customer satisfaction; (iii) employee satisfaction and (iv) new product or service development success. The Cronbach’s α for this scale was 0.817.

#### 3.2.5. Control variables

One-way ANOVA was performed to check significance difference across outcome variable. As per the results, at financial performance level, the authors found insignificance difference across gender (*F* = 2.64; *p* > 0.05), age (*F* = 1.92; *p* > 0.05), position (*F* = 0.44; *p* > 0.05) and significance difference across education (*F* = 4.06; *p* < 0.05), employee size (*F* = 6.70; *p* < 0.05) and ownership (*F* = 3.95; *p* < 0.05). At non-financial performance level, we found insignificance difference across gender (*F* = 3.11; *p* > 0.05), age (*F* = 0.81; *p* > 0.05), education (*F* = 2.58; *p* > 0.05), position (*F* = 1.62; *p* > 0.05), employee size (*F* = 0.82; *p* > 0.05) and ownership (*F* = 0.65; *p* > 0.05). Hence, we include education, employee size and ownership as controls on the model to test the hypotheses.

#### 3.2.6. Analytical strategy

The statistical software SAS9.4 and SmartPLS3.0 were used for data analysis. First, SAS9.4 was used for descriptive statistics. Then, SmartPLS3.0 was used to evaluate structural equation modelling. A PLS model is usually analyzed in two stages: (1) the assessment of the measurement model and (2) the assessment of the structural model. The Fornell-Larcker criterion, which stipulates that the square root of the average variance extract (AVE) of each latent construct should be higher than the correlation amongst the variables. As for the cross-loadings, all indicators loaded highest on their own scales. These two main measures mean that discriminant validity has been established (Fornell and Larcker, 1981). Furthermore, PLS-structural equation modelling path coefficients were estimated to test the hypothesized relationships.

## 4. Results

### 4.1. Demographics

This study included 211 managers (47.87% men and 52.13% women). Of the total, 20.85% of the respondents had a postgraduate degree, 73.94% had a bachelor degree and 5.21% had finished high school only. For age, 48.34% were under 30 years old, 35.07% were 31–35 years old, 13.75% were 36–40 years old and 2.84% were aged higher than 41 years. In the case of position, 6.64% were Chief Executive Officers (CEO)/general managers, 4.26% were senior vice-presidents, 35.55% were department managers and 53.55% were team managers. Furthermore, 24.64% of them worked for employers with fewer than 100 employees whilst 34.60% worked for employers with more than 2000 employees. Most of the companies belonged to private enterprises (46.45%). Moreover, the majority (86.73%) of the firms were from Zhejiang Province (see Table 1).

**Table 1:** Demographics.

Characteristic	Classification	Frequency	Percentage (%)
Gender	Male	101	47.87
	Female	110	52.13
Age	Under 30 years	102	48.34
	Between 31 and 35 years	74	35.07
	Between 36 and 40 years	29	13.75
	More than (and equal to) 41 years	6	2.84
Education	High school degree	11	5.21
	Bachelor degree	156	73.94
	Postgraduate degree	44	20.85
Position	Team manager	113	53.55
	Department manager	75	35.55
	Senior Vice-presidents	9	4.26
	CEO/General manager	14	6.64
Employee size	Fewer than 100	52	24.64
	Between 101 and 500	58	27.49
	Between 501 and 2000	28	13.27
	Between 2001 and 5,000	14	6.64
	More than (and equal to) 5,001	59	27.96
Ownership	Public	58	27.49
	Private	98	46.45
	Foreign-funded	16	7.58
	Joint ventures	17	8.05
	Other	22	10.43
Location of the company	Beijing	8	3.79
	Shanghai	8	3.79
	Zhejiang (Province)	183	86.73
	Anhui (Province)	12	5.69

### 4.2. The measurement model

Additionally, all item-factor loadings were greater than 0.5, with values ranging 0.593–0.926 (see Table 2), suggesting that the constructs exhibit satisfactory convergent validity (Hulland, 1999; Hall, 2008). All composite reliability values exceed the threshold of 0.7 and all AVE values exceed the threshold of 0.5. Further, all Cronbach’s alpha values were above the threshold of 0.6 (see Table 3), indicating good internal consistency (Fornell and Larcker, 1981). Moreover, PLS correlations and squared AVEs showed that the measurement model displayed good discriminant validity (see Table 4). Thus, statistical results of validity and reliability are adequate.

**Table 2:** Factor loadings from the PLS measurement model.

	Interactive use of PMS	Psychological empowerment	Creativity	Financial performance	Non-financial performance
pms1	**0.839**	0.141	0.336	0.293	0.317
pms2	**0.859**	0.170	0.333	0.282	0.361
pms3	**0.867**	0.172	0.307	0.358	0.381
pms4	**0.858**	0.208	0.287	0.210	0.229
pms5	**0.857**	0.151	0.346	0.265	0.352
emp1	0.152	**0.768**	0.530	0.234	0.359
emp2	0.136	**0.776**	0.522	0.238	0.377
emp3	0.174	**0.736**	0.505	0.307	0.350
emp4	0.176	**0.635**	0.283	0.161	0.228
emp5	0.191	**0.676**	0.312	0.102	0.239
emp6	0.168	**0.593**	0.277	0.180	0.211
emp7	0.254	**0.692**	0.304	0.255	0.219
emp8	0.094	**0.773**	0.373	0.180	0.267
emp9	0.069	**0.790**	0.367	0.192	0.284
emp10	0.103	**0.757**	0.339	0.191	0.235
emp11	0.127	**0.706**	0.377	0.229	0.310
emp12	0.105	**0.750**	0.378	0.285	0.304
ce1	0.292	0.552	**0.818**	0.393	0.488
ce2	0.281	0.513	**0.851**	0.387	0.522
ce3	0.373	0.501	**0.784**	0.348	0.459
ce4	0.333	0.457	**0.813**	0.361	0.500
ce5	0.230	0.490	**0.818**	0.343	0.445
ce6	0.316	0.368	**0.748**	0.230	0.413
ce7	0.264	0.508	**0.737**	0.298	0.503
ce8	0.314	0.439	**0.861**	0.396	0.587
ce9	0.374	0.412	**0.808**	0.382	0.590
ce10	0.297	0.441	**0.873**	0.405	0.633
ce11	0.311	0.378	**0.884**	0.389	0.584
ce12	0.309	0.396	**0.865**	0.435	0.608
ce13	0.323	0.420	**0.883**	0.386	0.603
fin1	0.342	0.310	0.466	**0.865**	0.675
fin2	0.278	0.255	0.349	**0.919**	0.578
fin3	0.237	0.250	0.365	**0.911**	0.554
fin4	0.302	0.263	0.409	**0.926**	0.615
nfin1	0.394	0.292	0.425	0.601	**0.712**
nfin2	0.341	0.345	0.523	0.614	**0.826**
nfin3	0.272	0.366	0.609	0.414	**0.851**
nfin4	0.235	0.280	0.507	0.590	**0.818**

The data in bold are the loading of an item. For validity of the discriminant, the loading should be stronger than their cross-loadings on other constructs. pms, interactive use of PMS; emp, psychological empowerment; ce, creativity; fin, financial performance; nfin, non-financial performance.

**Table 3:** Validity and reliability statistics.

Constructs	Average variance extracted	Composite reliability	*R* ^2^	Cronbach’s alpha	Communality
Interactive use of PMS	0.732	0.932	-	0.909	0.732
Psychological empowerment	0.523	0.929	0.040	0.917	0.523
Creativity	0.685	0.966	0.298	0.961	0.685
Financial performance	0.820	0.948	0.199	0.927	0.820
Non-financial performance	0.646	0.879	0.421	0.817	0.646
Global of fit of suggested model	0.362				

**Table 4:** Discriminant validity (Fornell-Larcker criterion).

Constructs	1	2	3	4	5
1. Interactive use of PMS	**0.856**				
2. Psychological empowerment	0.200	**0.723**			
3. Creativity	0.373	0.546	**0.828**		
4. Financial performance	0.325	0.301	0.446	**0.906**	
5. Non-financial performance	0.378	0.401	0.649	0.676	**0.804**

Diagonal elements represent the square root of the average variance extracted (AVE).

### 4.3. The structural model

We conducted a mediating analysis to examine the mechanism of how interactive PMS use affects performance *via* psychological empowerment and creativity. Bootstrapping method recommended by Edwards and Lambert (2007) was employed to test this mediation effect. PLS-path coefficient was used to test the hypotheses. Following the suggestion of Wetzels et al. (2009), we evaluated structural model fit using their calculation of goodness of fit. They derived the goodness-of-fit criteria as small: 0.1, medium: 0.25 and large: 0.36. Goodness of fit is defined as the geometric mean of the average communality and average R-square (for endogenous constructs). In Table 3, the global fit of the complete model was presented. As shown in Table 3, global fit was 0.362, indicating that the structural model has a good model fit.

**Figure 1:**
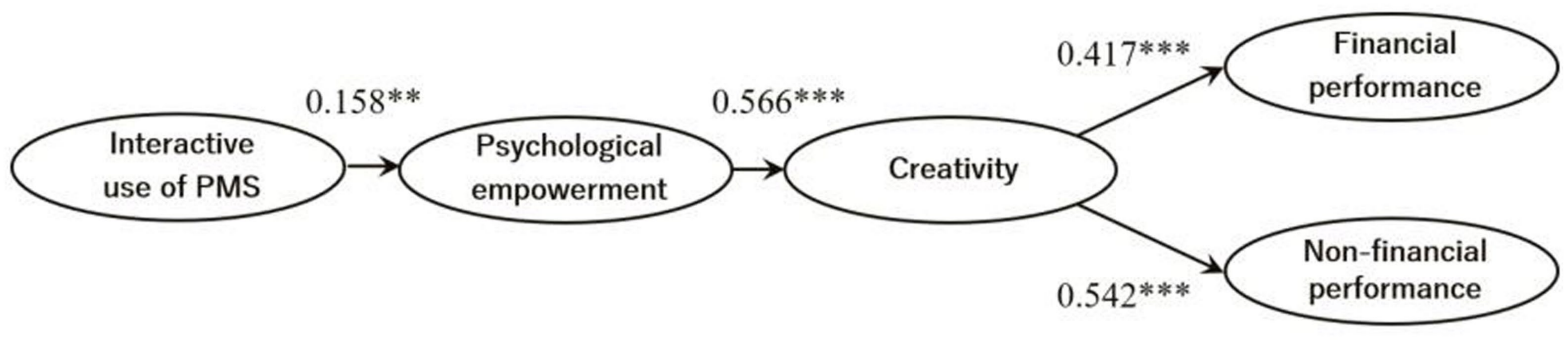
Final structural model. Paths of the control variables are omitted for clarity. Significant relationships are in bold. ***p < 0.01, **p < 0.05.

Table 5 presents the results of the PLS structural model. Figure 1 represents the final structural model. As expected, the interactive use of PMS was positively and significantly associated with psychological empowerment (ß = 0.158, *p* < 0.05). As such, hypothesis 1 (H1) was supported. Hypothesis 2 (H2) predicted a positive relationship between psychological empowerment and creativity. As expected, the association between psychological empowerment and creativity was positive and significant (ß = 0.566, *p* < 0.01). Therefore hypothesis 2 was supported. The result of bootstrapping also showed that creativity has a positive and significant effect on financial performance (ß = 0.417, *p* < 0.01) and non-financial performance (ß = 0.542, *p* < 0.01). Therefore hypothesis 3 was supported.

**Table 5:** Results of the PLS structural model.

Description of Paths	Path coefficient	*t*-value
Interactive use of PMS → Psychological empowerment	0.158	2.182^**^
Psychological empowerment → Creativity	0.566	8.962^***^
Creativity → Financial performance	0.417	5.179^***^
Creativity → Non-financial performance	0.542	9.429^***^

***p < 0.01, **p < 0.05.

Hypothesis 4 proposed that psychological empowerment mediates the association between the interactive use of PMS and creativity. Figure 1 shows that interactive use of PMS and psychological empowerment have a positive significant relationship, and psychological empowerment and creativity also have a positive and significant relationship. This means that psychological empowerment plays a significant mediating role between interactive use of PMS and creativity. It is difficult to increase creativity with only interactive use of PMS, and creativity can be expected to increase through psychological empowerment. Therefore, hypothesis 4 was supported.

Hypothesis 5 proposed that creativity mediates the association between psychological empowerment and performance. In this paper, performance is divided into two categories: financial and non-financial performance. Figure 1 shows that psychological empowerment has a positive and significant relationship with creativity. Creativity also shows a positive and significant relationship with both financial and non-financial performance. These results reveal that creativity plays a significant mediating role between psychological empowerment and performance. Psychological empowerment plays an important role in creativity in improving performance. Thus hypotheses 5, 5–1 and 5–2 were supported.

### 4.4. Tests for mediation

In existing literature, no unanimous answer exists on whether the relationship between independent and dependent variables must be significant, excluding the potential mediator (Zhao et al., 2010). The only requirement for mediation is that the indirect effect must be significant. If it is significant, the mediator absorbs some of the direct effect (Ali and Park, 2016). This study uses the non-parametric bootstrapping method to assess the significance of the mediation effect. Variance accounted for (VAF) is applied to calculate the indirect effect size in relation to the total effect (i.e., direct effect + indirect effect; Ali and Park, 2016). Table 6 shows that psychological empowerment as a mediator partially mediates the relationship between interactive use of PMS and creativity. Noticeably, the direct relationship between psychological empowerment and performance is not significant but their indirect effect is significant (ß = 0.236***, *t* = 4.474; ß = 0.307***, *t* = 6.370), leading to the conclusion that an indirect mediation between psychological empowerment and performance through creativity exists.

**Table 6:** Test of mediation by bootstrapping approach.

Effect of	Direct effect (*t*-value)	Indirect effect (*t*-value)	Total effect	VAF (%)	Interpretation	Conclusion
pms → emp → ce	0.263^***^ (4.671)	0.089^**^ (2.056)	0.352	25.28	Partial mediation	H4 supported
emp → ce → fin	0.103 (1.174)	0.236^***^ (4.474)	0.340	69.41	Indirect mediation	H5-1 supported
emp → ce → nfin	0.070 (1.142)	0.307^***^ (6.370)	0.376	81.65	Indirect mediation	H5-2 supported

***p < 0.01, **p < 0.05. VAF, variance accounted for. The VAF > 80% indicates full mediation, 20% ≤ VAF ≥ 80% shows partial mediation whilst VAF < 20% assumes no mediation (Ali and Park, 2016). pms, interactive use of PMS; emp, psychological empowerment; ce, creativity; fin, financial performance; nfin, non-financial performance.

## 5. Discussion

The study enhances our understanding of how the interactive use of PMS affects organizational performance. Based on data from 211 questionnaires collected from managers in Chinese firms, we empirically tested psychological empowerment and creativity as mediators of the influence of interactive PMS use on organizational performance. The results show positive significant association between interactive PMS use and psychological empowerment, psychological empowerment and creativity, as well as creativity and organizational performance. Additionally, the results show that interactive PMS benefits creativity *via* psychological empowerment. Furthermore, creativity mediated the association between psychological empowerment and organizational performance.

Results (H1) demonstrate that interactive PMSs capture the benefits of individual initiative. The interactive use of PMSs provides adequate performance information, stimulates dialogue and learning to build responsive organizations and thus improves psychological empowerment. Performance information generated by interactive PMS is interpreted and discussed in face-to-face meetings (Simons, 1995) which ultimately empowering managers psychologically. This has been acknowledged by Hall (2008). Specifically, the information provided and shared by interactive PMS use is necessary for managers to develop the psychological experience of empowerment. These findings are also complementary to the empirical research conducted by Marginson et al. (2014) that support the interactive use of PMS as crucial to increasing psychological empowerment.

Furthermore, results (H2) also indicate that psychological empowerment is an important factor for achieving creativity. Psychological empowerment is defined as ‘a motivational construct manifested in four cognitions: meaning, competence, self-determination, and impact’ (Spreitzer, 1995). In a work context, the perception of psychological empowerment has great potential to boost individual creativity because it increases concentration, self-efficacy and initiative. Consistent with Moulang (2015) and Speklé et al. (2017), these findings also show that a more psychological experience of empowerment fosters high levels of individual creativity. Additionally, results (H3) indicate a significant effect of creativity on organizational performance. Our findings demonstrate that individual creativity, which is a primary capability within the firm, leads to increased competitive advantage and enhanced performance. Consistent with Im and Workman (2004), the results also indicate that creativity is an important determinant of an enterprise’s performance. Additionally, this finding also extends the work of Gong et al. (2009) by empirically testing the effect of individual-level creativity on financial/non-financial performance gains.

Results (H4) indicate that psychological empowerment does serve as a mediator between interactive PMS use and creativity. Since interactive PMSs provide adequate performance information, they also create a responsive climate that fosters psychological empowerment (Simons, 1995), and thus enhances individual creativity. Using a control system interactively can activate an individual’s cognition and motivation, and lead to the proposal of novel ideas. This finding is in accordance with Moulang (2015), who showed that interactive PMS can benefit individual creativity by improving psychological empowerment. Furthermore, this result also complements the studies conducted by Franco-Santos et al. (2012), who calls for research to focus on the consequences of PMS on an individual’s behavior. Hence, this evidence suggests that managers’ psychological empowerment and creativity are improved through the usage of interactive PMS.

Results (H5) demonstrate that psychological empowerment positively affects organizational performance by enhancing creativity. To facilitate creativity and improve organizational performance gains, a high level of psychological empowerment should be fostered within the firm. We have concluded that managers’ creativity is a critical factor of improving organizational performance. Our finding is consistent with the arguments developed by Caniëls et al. (2014), who claim that psychological empowerment cognitions help individuals to satisfy innate desires to create, which, in turn, improves a firm’s performance gains (Weinzimmer et al., 2011; Sung and Nam, 2012). Therefore, perceptions of psychological empowerment help reinforce a sense of self-determination, competence and dexterity, which, in turn, boost individual creativity and ultimately contribute to organizational performance.

### 5.1. Theoretical contributions and practical implications

This study contributes to the literature exploring the relationship between PMS and performance (e.g., Henri, 2006b; Harrison et al., 2022) by analysing the role of psychological empowerment and creativity. The findings suggest that both psychological empowerment and creativity help explain the association between interactive PMS and organizational performance. We have identified the role of interactive PMS use in the relationships between psychological empowerment, creativity and performance, thereby improving the scholarly understanding of the theoretical value of PMS in relation to individual’s behavior and performance consequences. The findings show that interactive PMS use, psychological empowerment and creativity jointly influence organizational performance. More specifically, the interactive use of PMS cultivates organizational performance by not only strengthening the perceptions of psychological empowerment but also individual-level creativity. Interactive PMS use affects organizational routines by influencing the way managers behave. To remain competitive, organizations and managers should shape a bottom-up process, and facilitate the communication of organization participants, whilst monitoring the impact of interactive controls on individual’s behavior and performance outcomes.

Second, this study expands on the literature testing PMS and psychological empowerment according to cognitive-based psychology theory. The findings confirm Franco-Santos et al.’s (2012) results that interactive PMS can positively influence the behavior of managers. These results suggest that psychological empowerment is boosted when managers use PMS interactively at different levels of firms. Our findings support prior research (Franco-Santos et al., 2012; Akroyd and Kober, 2020), which holds the view that the design and use of PMS are crucial for managers’ behavior and organizational outcomes. Additionally, our research addresses Moulang’s (2015) call for further study to refine and validate the creativity instrument. This study differs from the work of Moulang (2015) in which the creativity measurement is derived from three different studies. We also use Zhou and George (2001) scale to measure creativity. The findings show that the instrument is a reliable and valid measurement of the creativity construct.

Furthermore, this study contributes to PMS literature by exploring relationships using a Chinese sample. Most researchers have examined the effects of PMS using companies located in Western countries. However, prior research (e.g., Koufteros et al., 2014; Guenther and Heinicke, 2019) suggests that empirical data can be applied to a different geographical area. Expanding upon prior research, we also included organizational outcomes in the research model. Our findings demonstrating that the extent of interactive PMS use is indirectly associated with organizational performance through empowerment and creativity expand upon Appuhami (2019) and Hall (2008) empirical findings. Finally, these findings expand upon the work of Harrison et al. (2022), which is based on survey data from senior managers in organizations. Compared with earlier theoretical and empirical work (e.g., Hall, 2008; Harrison et al., 2022), we simultaneously broadened the nature of the sample, indicating that interactive use of PMSs is beneficial for psychological empowerment at all levels of managers. Consequently, the study highlights the importance of interactive PMS use in achieving higher levels of individual behavior. It also suggests that managers should collect and generate adequate performance information through interactive controls to improve the motivation of organization members.

In practical terms, our results suggest that managers should engage in an interactive dialogue with their subordinates and peers, to meet their needs and provide constructive feedback, so that organization participants feel empowered and valued. The sense of managers’ psychological empowerment is positively affected through interactive control systems. Interactive PMSs create a responsive firm that cultivates the sense of an individual’s psychological empowerment. Our study highlights that interactive control systems play a critical role in shaping a participative climate (Simons, 1995) and that such systems improve the cognitions of self-efficacy through providing performance information, facilitating organization members’ trust and enhancing their ego involvement. Therefore, to strengthen organization participants’ psychological empowerment in the workplace, managers should schedule face-to-face meetings regularly, stimulate conversation and share their thoughts, to help them better understand the meaning of what they do.

Our results have also shown that interactive PMS use has a significant influence on creativity *via* psychological empowerment. Our findings suggest that the interactive use of PMSs can monitor changing external environments, stimulate dialogue throughout the organization, encourage individuals to seek, generate and test new ideas, and thus gain a competitive advantage. Additionally, creativity is also an impact factor that influences organizational performance. This also suggests that creativity is a distinctive resource within the firm that can effectively improve performance. To create a higher performance firm, organizations should not only look at utilising the multiplicity and variety of information contained in the PMS but also at facilitating and triggering individuals’ intrinsic task motivation and creativity. In sum, we suggest that managers should invest in building an interactive PMS to provide a comprehensive performance information platform that facilitates intrinsic motivation encouraging individuals to develop novel and useful ideas. This will further contribute to an overall improvement in financial and non-financial performance.

### 5.2. Limitations and future research

The findings have several limitations. First, the study’s generalizability is slightly limited in the sense that data were collected only from companies in an economically advanced region of China. Future studies could collect data from other areas of China or elsewhere to improve the applicability of our results. Second, our findings suffer from the limitations of cross-sectional design. Thus, case studies or longitudinal data could be further used to conduct a formal test of causality. Survey Data collected from small-size organizations were also included, which may affect the causal relationship. Additionally, the subjective bias inherent in questionnaire research is also a limitation of the study. Third, whilst our measurement scale for psychological empowerment is based on a holistic view, psychological empowerment does comprise four components (Spreitzer, 1995), so the link between other aspects of psychological empowerment, interactive PMSs and creativity should be classified further in future research. Finally, contextual variables such as environmental uncertainty should be further included to develop a more comprehensive theoretical model.

Despite these limitations, the current study has several strengths. First, existing studies have focused only on strategic business unit (SBU) managers. However, our conclusions were derived from all managerial levels. Second, more specifically, this study investigates the role of interactive PMS use to extend the current PMS research. Particularly, we examined a wider range of effects that PMS has on individual behavior by testing the linkage between PMSs, psychological empowerment and creativity. Third, we have broadened the theoretical base of investigating creativity as a mediator between psychological empowerment and organizational performance.

## 6. Conclusion

The aim of this study was to determine whether and how PMS use affects performance by testing the mediation effects of psychological empowerment and creativity. To examine our hypotheses, we conducted empirical study with 211 managers from Chinese firms. Our results revealed that (1) the interactive use of PMS enhances organization participants’ psychological empowerment; (2) psychological empowerment positively influences creativity; (3) higher levels of creativity improve organizational performance; (4) the relationship between interactive PMS use and creativity is mediated by psychological empowerment and (5) creativity has a mediating effect between psychological empowerment and organizational performance.

## Data availability statement

The raw data supporting the conclusions of this article will be made available by the authors, without undue reservation.

## Ethics statement

The studies involving human participants were reviewed and approved by the Institutional Review Board of Business School, Ningbo University. The patients/participants provided their written informed consent to participate in this study.

## Author contributions

LZ and DK designed and performed the research. LZ collected and analyzed the data. SD and LZ wrote the manuscript. All authors read and approved the final manuscript.

## Funding

This study was supported by the Academy of Longyuan Construction Financial Research Grant (Grant Number: LYZDB2004) and Ningbo University High-level Humanities and Social Sciences Cultivation Project (Grant Number: XPYQ22002).

## Conflict of interest

The authors declare that the research was conducted in the absence of any commercial or financial relationships that could be construed as a potential conflict of interest.

## Publisher’s note

All claims expressed in this article are solely those of the authors and do not necessarily represent those of their affiliated organizations, or those of the publisher, the editors and the reviewers. Any product that may be evaluated in this article, or claim that may be made by its manufacturer, is not guaranteed or endorsed by the publisher.
